# Supercapacitors: An Efficient Way for Energy Storage Application

**DOI:** 10.3390/ma17030702

**Published:** 2024-02-01

**Authors:** Mate Czagany, Szabolcs Hompoth, Anup Kumar Keshri, Niranjan Pandit, Imre Galambos, Zoltan Gacsi, Peter Baumli

**Affiliations:** 1 Institute of Physical Metallurgy, Metal Forming and Nanotechnology, University of Miskolc, 3515 Miskolc, Hungary; szabolcs.hompoth@uni-msikolc.hu (S.H.); zoltan.gacsi@uni-miskolc.hu (Z.G.); 2 Plasma Spray Coating Laboratory, Metallurgical and Materials Engineering, Indian Institute of Technology Patna, Bihta 801106, Bihar, India; anup@iitp.ac.in (A.K.K.); karthik.niranjan01@gmail.com (N.P.); 3 Asianet Hungary Ltd., 1033 Budapest, Hungary; galambos.imre01@gmail.com

**Keywords:** supercapacitor, energy storage, EDLC, pseudocapacitance, electrode, electrolyte

## Abstract

To date, batteries are the most widely used energy storage devices, fulfilling the requirements of different industrial and consumer applications. However, the efficient use of renewable energy sources and the emergence of wearable electronics has created the need for new requirements such as high-speed energy delivery, faster charge–discharge speeds, longer lifetimes, and reusability. This leads to the need for supercapacitors, which can be a good complement to batteries. However, one of their drawbacks is their lower energy storage capability, which has triggered worldwide research efforts to increase their energy density. With the introduction of novel nanostructured materials, hierarchical pore structures, hybrid devices combining these materials, and unconventional electrolytes, significant developments have been reported in the literature. This paper reviews the short history of the evolution of supercapacitors and the fundamental aspects of supercapacitors, positioning them among other energy-storage systems. The main electrochemical measurement methods used to characterize their energy storage features are discussed with a focus on their specific characteristics and limitations. High importance is given to the integral components of the supercapacitor cell, particularly to the electrode materials and the different types of electrolytes that determine the performance of the supercapacitor device (e.g., storage capability, power output, cycling stability). Current directions in the development of electrode materials, including carbonaceous forms, transition metal-based compounds, conducting polymers, and novel materials are discussed. The synergy between the electrode material and the current collector is a key factor, as well as the fine-tuning of the electrode material and electrolyte.

## 1. Introduction

The increasing utilization of clean renewable energy sources [1,2] implies a need for the development of different energy storage technologies. The main goal of this development is to reduce and slowly eliminate the economic and ecological drawbacks of using conventional sources of energy. Energy storage technology is a key factor to manage the revolving nature of renewable energies and to meet the energy needs of rapidly evolving electronic devices and electric vehicles [3,4]. Electrochemical energy, supported by batteries, fuel cells, and electrochemical capacitors (also known as supercapacitors), plays an important role in efficiently supporting the required modern energy demands. The electrochemical properties of these devices are very similar; however, their energy storage and conversion mechanisms are different [5,6]. Supercapacitors (SCs) have gained much attention due to their high specific capacitance, fast storage capability, and long life cycle. An SC is used as a pulse current system to provide a high specific power (10,000 W/kg) and high current for the duration of a few seconds or minutes [7,8]. They can be used alone, or in combination with another energy storage device (e.g., battery) to for their efficient application in a wide range of fields, including consumer electronics, hybrid electric vehicles, solar energy production, and industrial power management [9]. Furthermore, supercapacitors are recyclable and have a much longer lifespan compared to batteries, thereby meeting the expectations of an environmentally friendly future.

The main drawback of SCs is that they are unable to store as much energy as a conventional rechargeable battery. Thus, research efforts usually aim to increase the energy storage capacity of SCs, with a focus on developing newly designed electrodes.

The recent publications [10,11,12,13,14] have typically focused on a specific group of materials, and provided information on the current scientific knowledge, the most typical physicochemical properties of the given materials, their supercapacitive behavior, and the production methods of these materials. This review provides an introduction to the fundamental aspects of SCs, while presenting a comprehensive overview of current directions in the development of the components of SCs in the following structure: Section 2 provides a brief history of the evolution of SCs, giving an insight into the different stages of their development. In Section 3, the main concepts and attributes of SCs are discussed, including the classification of SCs. Section 4 describes the main electrochemical measurement methods used to characterize the performance and energy storage capabilities of SCs. Section 5 summarizes the main components of SCs and presents possible material solutions tailored to the performance requirements of SCs.

## 2. Short History of Supercapacitors

During our early history, the idea of storing electrical charges on surfaces stemmed from a phenomenon associated with amber friction, observed in ancient Greece [15]. However, it was only in the 19th century that electricity was understood at the molecular level, starting with the work of Michael Faraday and later that of J.J. Thomson and Millikan on electrons [3]. Another milestone was the development of an electronic device named the Leyden jar [16] by Pieter van Musschenbroek, who discovered the charge separation and charge-storage principle. The Leyden jar was referred to as “Condenser”, and later on as “Capacitor”.

**Figure 1:**
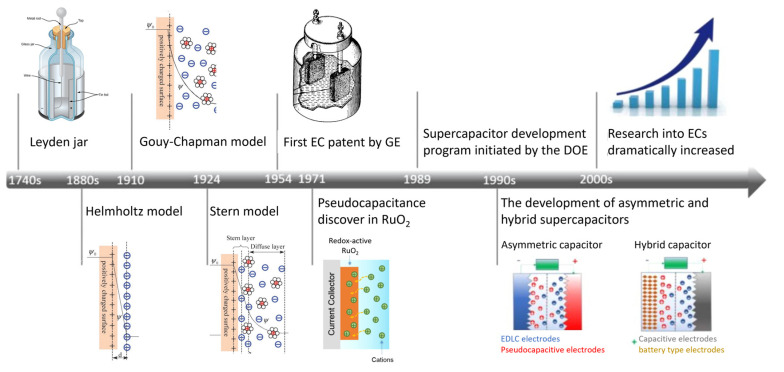
Timeline of the development of SCs, reproduced with permission from reference [34]. Copyright (2018) American Chemical Society.

The first concept for an electric double layer (EDL) was proposed by Helmholtz in the 19th century (Figure 1). Based on the model, opposite charges are layered at the electrode/electrolyte interface, parted by only an atomic distance, similarly to a two-plate conventional capacitor. The model was later modified by Gouy and Chapman (Figure 1), who proposed a diffusive layer. The model suggests that the same amount of opposite charge appears in the electrolyte, surrounding the charged solid surface, but the ions are not rigidly attached to the surface; rather, a diffused layer is formed. The thickness of the diffuse layer is partly determined by the kinetic energy of the ions in the electrolyte [17]. Stern combined these two models and proposed a model with two regions of the particle distribution: an inner region (Stern layer, compact layer) and a diffusive layer (Figure 1). The model states that the ions have a finite size, thereby limiting their approach to the surface. The Stern layer consists of surface-adsorbed ions and contains two planes: specifically adsorbed ions (forming the inner Helmholtz plane: IHP) and non-specifically adsorbed counter-ions (outer Helmholtz plane: OHP). The diffusive layer region is explained by the Gouy–Chapman model: the kinetic energy of the counter-ions results in a diffusive layer, affected by the thickness [3,18].

The idea of storing a charge in an EDL was patented in the 1950s [19]. It described the notion that charges form a double layer in the interface between solid material and the electrolyte. A decade later, Standard Oil Company in Cleveland committed to investing in this technology, and carried out a significant amount of development in this field, but due to a lack of sales, the further development of this technology was put on hold, and eventually licensed out to Nippon Electric Company (NEC, Tokyo, Japan) in the 1970s [20]. NEC started to produce low-powered devices for memory backup under the name “Supercapacitor” [21]. Later on, other companies started to adapt this project, with different names for these devices such as “Panasonic Gold Capacitor”, a name from Matushita in 1978 [22], or “Dynacap”, a name given by ELNA in 1987 [23].

Pseudocapacitors, a new class of electrochemical capacitors based on RuO_2_, were discovered in 1971. The discovered pseudocapacitance opened up the ability of storing larger amounts of charge within these devices [24,25]. The first time a high-power, double-layer capacitor was produced was in the beginning of the 1980s, developed by the Pinnacle Research Institute, and was called an “Ultracapacitor”. Later on, the Department of Energy in the United States started to study the concept in the context of hybrid electric vehicles [26]. After the United States began investing in its research, Maxwell laboratories, in the beginning of the 1990s, began making different types of SCs available, e.g., electric double layer capacitors (EDLCs), pseudocapacitors, and asymmetric supercapacitors [26].

**Figure 2:**
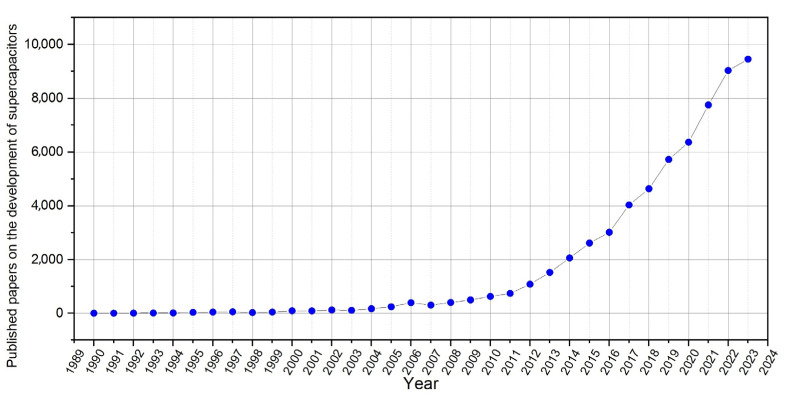
Timeline of papers written on the development of SCs according to Science Direct (www.sciencedirect.com, accessed on 12 January 2024).

Today, several companies produce supercapacitors, such as Nippon Chemi-con, KEMET (Yageo Company, Fort Lauderdale, FL, USA), SPSCAP, etc., and even more researchers are working to expand the knowledge in this field of study (Figure 2), as well as to summarize the latest results and the current state of understanding [27,28,29,30] to create a more sustainable and efficient future, such as by providing solutions like the Supercapacitor-powered buses in China [31], Hong Kong [32], and South-Korea [33].

## 3. Fundamentals of Supercapacitors

### 3.1. Principles and Properties

**Figure 3:**
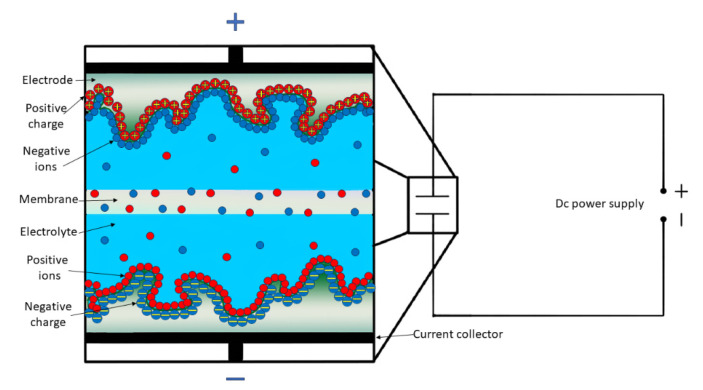
Schematic of a SC cell.

Supercapacitors are basically electrochemical cells, comprising two electrodes (anode, cathode), an electrolyte, and a separator (Figure 3). The electrodes are usually porous structured materials which are separated by an ion-permeable membrane. For symmetric cells, the electrodes can be identical, while they can be different for asymmetric and hybrid cells [35,36]. The basic operation of a SC is the following: when charging, the electrons are forced to move from the positive electrode to the negative electrode trough an external circuit. Consequently, the cations in the electrolyte concentrate in the vicinity of the negative electrode, while anions do the same in the positive electrode, forming an EDL or inducing Faradaic redox reactions to compensate for the external charge unbalance. During discharge, the electrons travel from the negative to the positive electrode through the external circuit, resulting in the mixing of both kinds of ions in the pores or inducing the reverse of the Faradaic reactions.

A supercapacitor is able to provide a-hundred-times-higher power than batteries in the same volume, although the amount of charge it can store is usually 3–30 times lower. Thus, they are suited perfectly for applications that need a large amount of power in a very short time, i.e., power bursts, but for which high energy storage capacity is not essential [37,38,39]. Conventional capacitors (electrostatic, electrolytic) store charge on relatively low-surface-area plates, while SCs store their charge either in an electric double layer set up by ions or by fast redox reactions taking place at the interface between an electrode with a high specific surface area (SSA) and a liquid electrolyte [40].

**Figure 4:**
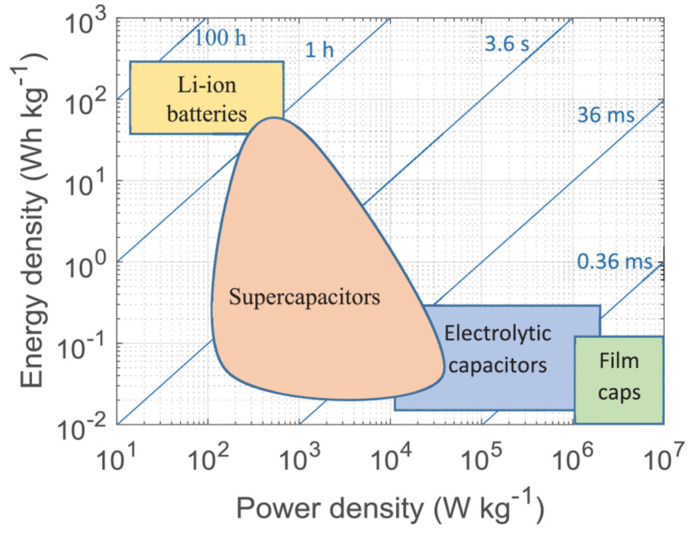
Ragone plot of different energy storage technologies [36]. Licensed by CC BY 4.0 (http://creativecommons.org/licenses/by/4.0, accessed on 12 January 2024).

Figure 4 shows a Ragone plot that represents the related power and energy densities, which can be used for comparing various energy-storing devices. As can be seen, SCs are situated between conventional electrolytic capacitors and batteries. The power output of a SC is lower than that of an electrolytic capacitor (can still exceed 1–10 kW/kg), but their specific energy can be several orders of magnitude higher. Batteries, on the other hand, are capable of storing large amount of energy but, as a consequence of their storage mechanism, they offer lower power densities. The unique combination of a high power output and good specific energy allows SCs to occupy a functional position between batteries and conventional capacitors [9,41,42]. The discharge times of the different devices are also shown in Figure 1 as diagonal lines. Capacitors take only a split second or a few seconds to discharge, while batteries usually take longer time. As can be seen, SCs can also be charged in a reasonable time, usually lower than 1 h. In order to get a full picture of the advantages and limitations of the different energy storage technologies, other important parameters also need to be taken into account beside the Ragone plot, such as life cycle, cost, and safety [43,44].

**Figure 5:**
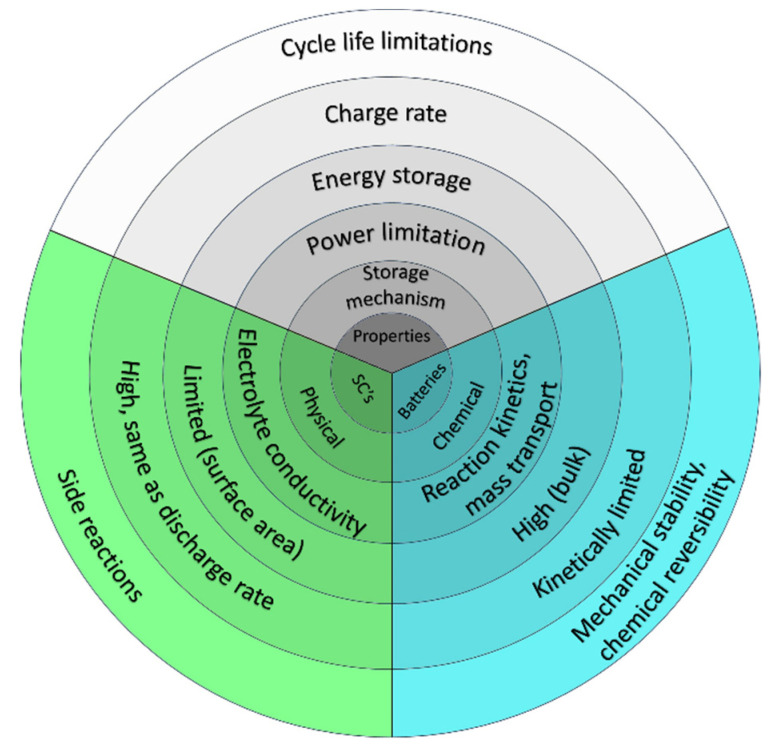
Comparison of properties of rechargeable batteries and electrochemical capacitors [4].

The cycle life of SC devices is another great advantage, as they can be operated with from 100,000 to 1 million cycles, owing to their charge-storage mechanism. They can store charges physically at the surface of the electrodes without irreversible chemical reactions, thereby exceeding the cycle life of batteries. Batteries are limited by the swelling during the typical redox reactions in the bulk of the active material during the charging and discharging processes. However, in the case of the majority of SCs, there are no changes in the electrode volume due to their highly reversible electrostatic storage. Furthermore, the simple charge-storage mechanism generates less heat compared to batteries, which allows safer, reliable use [45]. Figure 5 compares the differences between the properties of batteries and electrochemical capacitors.

The working temperature range of different energy storage devices is also important to mention. With the use of SCs, high power performance down to −40 °C can be achieved, which is usually not possible for batteries. Furthermore, SCs are considered to be safer than batteries for processes of high-power-rating charging and discharging [4,46].

### 3.2. Classification of Supercapacitors

**Figure 6:**
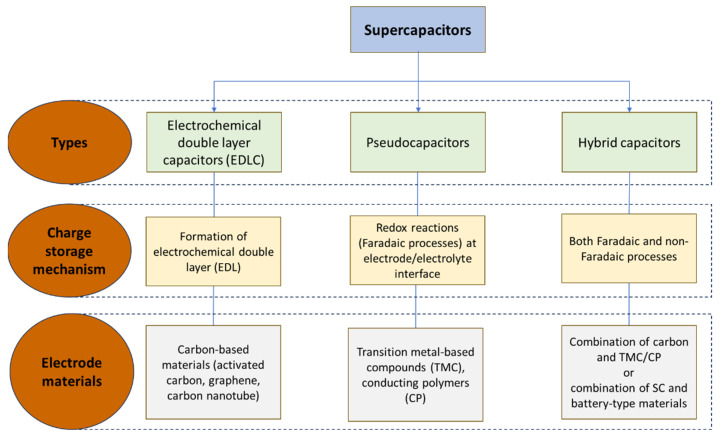
Classification of supercapacitors.

In terms of their applicability, one of the most important aspect of SCs is their energy storage mechanism, as their analysis and evaluation can be misinterpreted if the applied electrodes of a SC device are made of different materials [36]. Depending on the storage mechanism, SCs can be classified as electric double-layer capacitors (EDLCs), pseudocapacitors (PCs, also known as redox capacitors), and hybrid capacitors (Figure 6).

#### 3.2.1. Electric Double Layer Capacitors (EDLCs)

Electric double layer capacitors store energy the same way as traditional capacitors, i.e., by charge separation. The double layer consists of negative (anion) and positive (cation) charges accumulated and electrostatically stored at the interface between the electrode and the electrolyte. EDLCs are based on nanoporous active electrode materials with a high specific surface area of >1000 m^2^/g. These nanoporous materials are usually based on carbon, owing to its low cost, availability, and easy production methodology [47]. In comparison with conventional capacitors, the highly extended surface area of the electrode enables EDLCs to store an increased amount of charge, thus leading to higher capacitance values. As the surface density of the charges depends on the applied voltage, the capacitance of the electrode varies with the electrode potential. The only electrochemical reaction taking place is the adsorption and desorption of the ions at the surface of the electrode, enabling an intrinsically rapid energy storage mechanism [36].

#### 3.2.2. Pseudocapacitors

As mentioned earlier, the pseudocapacitive mechanism was recognized in the 1970s, and is based on fast redox reactions taking place only at or near the electrode surface, not like the bulk of batteries [41,48]. This behavior has EDLC-like electrochemical features but usually holds a significantly higher specific capacitance [49]. Three main types of pseudocapacitance can be distinguished: adsorption pseudocapacitance, defined by monolayer adsorption (e.g., Pt electrode [50]); redox pseudocapacitance, which is based on faradaic reactions (e.g., hydrous RuO_2_ [51]); and intercalation pseudocapacitance, provided by solid–solution electrochemical intercalation (e.g., Nb_2_O_5_ [52]). The extent of the reaction (determined by surface coverage, surface redox, intercalation) depends almost linearly on the potential (V) in each of these types [53]. One of the most promising is the intercalation type, which stores charge in the bulk of the electrode material via the rapid intercalation/deintercalation of ions, thus bridging the gap between conventional batteries and SCs [54,55].

In addition, different nitrides, carbides, and conducting polymers can also be applied, combining electrostatic and pseudocapacitive charge-storage mechanisms [56,57,58]. One of their disadvantages, however, is their lower life cycle, as redox reactions can lead to poor mechanical stability due to the swelling/shrinking of the electrodes. Another drawback is their lower power density (P_d_) as a consequence of the involved reaction dynamics [53].

#### 3.2.3. Hybrid Capacitors

The third group, hybrid capacitors, combine the properties of the first two, having an EDLC electrode and a pseudocapacitive or battery-type electrode. The concept of a hybrid construction came from the effort to increase the energy density (E_d_) of SCs to an interval of 20–30 Wh/kg [59]. In their case, one-half of the hybrid SC cell acts as an EDLC while the other half behaves as a pseudocapacitor/battery. In this way, SCs with a low-cost, good electrical conductivity, mechanical flexibility, and chemical stability can be obtained. The combination of the two types of electrodes leads to overshadowing the limiting properties of the individual electrodes, thereby enabling the use of higher working potentials and exhibiting a higher specific capacitance (usually two to three times more) in comparison with EDLCs and pseudocapacitors. Hybrid capacitors can be further divided in three groups, as asymmetric hybrid, battery type hybrid, and composite hybrid SCs [60,61,62].

## 4. Characterization of Supercapacitive Behavior

**Figure 7:**
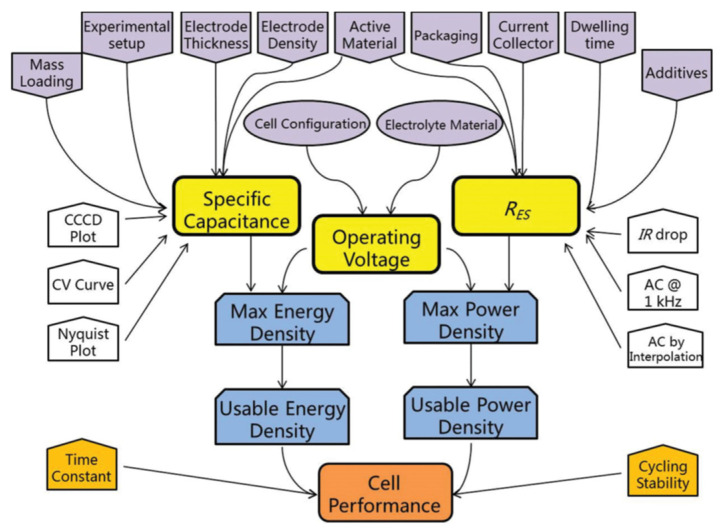
The major factors affecting supercapacitive performance. Reprinted (adapted) with permission from [63]. Copyright Wiley-VCH GmbH.

The three core parameters required to efficiently characterize the energy storage capability and power performance of SCs are total capacitance (C_T_), operating voltage (V_o_), and equivalent series resistance (R_ES_ or ESR). However, in the research sector, there are other factors that are essential to developing different electrode materials and new cell designs, such as the energy and power density, time constant, cycling stability, and operating voltage range. The complex relations between the different factors are presented in Figure 7. Three techniques are generally used to characterize the supercapacitive behavior of SCs: cyclic voltammetry, galvanostatic charge–discharge, and electrochemical impedance spectroscopy. Their main objective is to evaluate the electrochemical characteristics of energy storage systems from different points of view. These methods generally measure three basic parameters, current, voltage, and time, while the other parameters can be calculated from them. However, due to the different mechanisms of the measurement techniques, the results may appear contradictory; thus, it is essential to indicate the applied parameters alongside the obtained results [63,64].

Cyclic voltammetry (CV) measures current using a fixed scan rate, while changing the potential and galvanostatic charge/discharge (GCD) use a constant current density to reach the desired potential values and electrochemical impedance spectroscopy (EIS) uses impedance or capacitance. The main features of the measurement methods are summarized in Table 1. Among these methods, GCD is considered to be the most reliable parameter for evaluating the electrochemical behavior of SCs [65,66].

### 4.1. Cyclic Voltammetry

**Figure 8:**
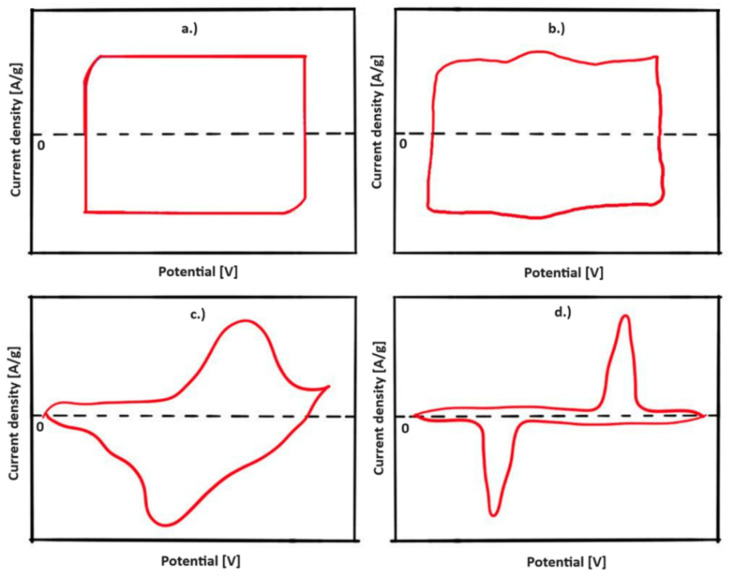
Schematic cyclic voltammograms of different charge-storage mechanisms. (**a**) EDLC behavior, (**b**) surface redox pseudocapacitance, (**c**) faradaic dominated pseudocapacitance, (**d**) typical battery behavior.

In the case of CV, the potential is linearly changed between the working and counter electrodes within a two-electrode system, or between the working and reference electrode for three-electrode systems. During the measurement, the current induced by the potential change is recorded. The scan rate represents the speed of the potential change (V/s), while the potential window is the potential range in which the measurement is performed [42]. The diagrams provided by CV are voltammograms (Figure 8). Their shape allows identification of the types of electrochemical reactions, i.e., it differentiates between EDLC, pseudocapacitive, and battery-like behavior. The shape is usually rectangular for EDLC (Figure 8a) and, for most pseudocapacitive materials, reversible redox peaks might be superimposed on the pseudocapacitive curves (Figure 8b,c) [49,67]. The potential window, and the reversibility of anodic and cathodic processes, can also be studied by this method.

The specific capacitance of the electrode material, or the SC cell, can be calculated from a cyclic voltammogram by the following equation:

(1) $$C_{s}=\frac{\int_{V1}^{V2}\left|I(V)\right|\;dV}{2\cdot \Delta V\cdot v\cdot \Pi }$$

where $\int_{V1}^{V2}\left|I(V)\right|\;dV$ is the integrated area of a cycle of a CV curve, *v* is the scan rate ($\frac{V}{s}$), ΔV is the potential range of one CV cycle (V), and Π can indicate the mass, surface area, or volume of the electrode material, or even the size of the electrode.

The capacitance of an SC usually changes with the applied scan rate. Tanwilaisiri et al. [68] found that the capacitance of a 3D printed, activated, carbon-based SC decreased from 182 mF to 32 mF as the scan rate increased from 0.02 to 0.1 V/s. Similarly, a decrease in the capacitance of electroless Ni-B coatings with pseudocapacitive properties from 30.61 to 24.61 mF/cm^2^ was observed when increasing the scan rate from 0.01 to 0.1 V/s [69]. The decrease can be attributed to a reduced ion diffusion process as, at lower rates, ionic diffusion has enough time to penetrate the inner pores of the electrode.

### 4.2. Galvanostatic Charge-Discharge

**Figure 9:**
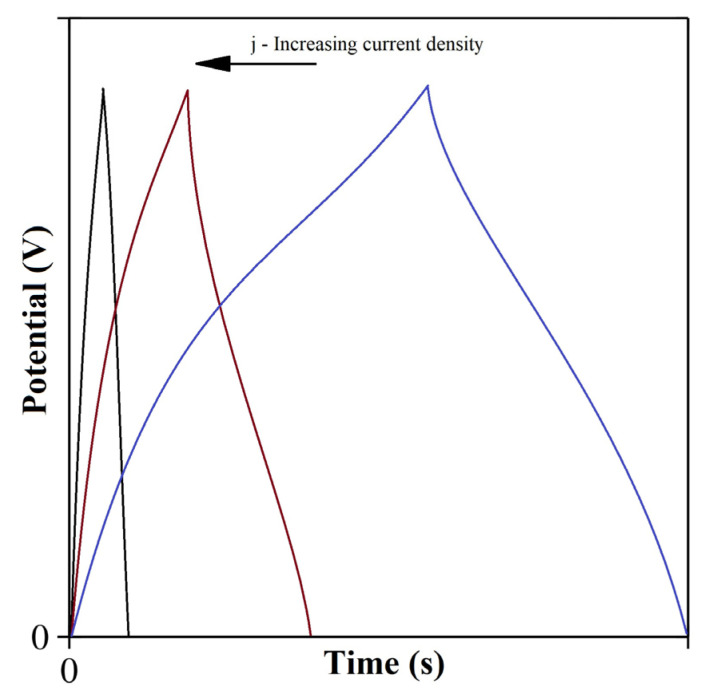
Schematic representation of galvanostatic charge–discharge (GCD) diagram.

During galvanostatic charge–discharge (GCD) measurement, successive charging–discharging processes can be performed at a fixed, constant current value (Figure 9). With this method, a supercapacitive electrode (as working electrode) or a complete SC device can also be measured. GCD is traditionally used to evaluate several SC performance parameters: capacitance, energy density, power density, R_ES_, and cycle stability. Its most important use is checking the stability of SCs [63,70]. From the results of GCD measurement, the specific capacitance can also be calculated:

(2) $$C_{s}=\frac{I\cdot \Delta t}{\Pi \cdot \Delta V}=\frac{I\cdot \int_{t_{V1}}^{t_{V2}}V\left(t\right)dt}{{\left(\Delta V\right)}^{2}\cdot \Pi }$$

where *I* is the constant current, *Δt* is the charging or discharging time, *ΔV* is the specified potential change, and $\int_{t_{V1}}^{t_{V2}}V\left(t\right)dt$ is the integrated area of the charging or discharging curve. The specific energy and power density values can also be calculated from the results of GCD measurements. The stored energy can be calculated from the charging curve, while the energy to be delivered, and thus the power density, can be obtained from the discharging curve by the following equations:

(3) $$E_{D}=\frac{1}{2}\cdot C_{S}\cdot {\Delta V}^{2}=\frac{I\cdot \int_{t_{Vmax}}^{t_{Vmin}}V\left(t\right)dt}{2\cdot \Pi }$$

(4) $$P_{D}=\frac{E_{D}}{\Delta t}=\frac{I\cdot \int_{t_{Vmin}}^{t_{Vmax}}V\left(t\right)dt}{2\cdot \Delta t\cdot \Pi }$$

where *t_V(max)_* is the ending time of a charge or the starting time of a discharge during one cycle (s), *t_V(min)_* is the starting time of a charge or the ending time of a discharge in one cycle (s), and Δt is the charge/discharge time.

Cao et al. [71] found that the specific capacitance (C_S_) of ionic-liquid-doped polymer nanocomposite capacitors could be as high as 520 F/g at a current density of 0.5 A/g, while the energy and power densities were calculated as 58.5 Wh/kg and 22.5 W/kg, respectively. They reported that 82% of the capacitance was retained after 800 field cycles at 3A/g. Hou et al. [72] checked the performance of porous polypirrole films using GCD. The C_S_ and E_d_ were 286 F/g and 39.7 Wh/kg, respectively, at 0.5 A/g, while 86% of the initial capacitance was retained after 3000 cycles. Even though GCD is appropriate for evaluating electrical properties, its disadvantage is that it shows the same triangular shape between the cell voltage and time for all double-layer capacitive (DLC) materials.

### 4.3. Electrochemical Impedance Spectroscopy

**Figure 10:**
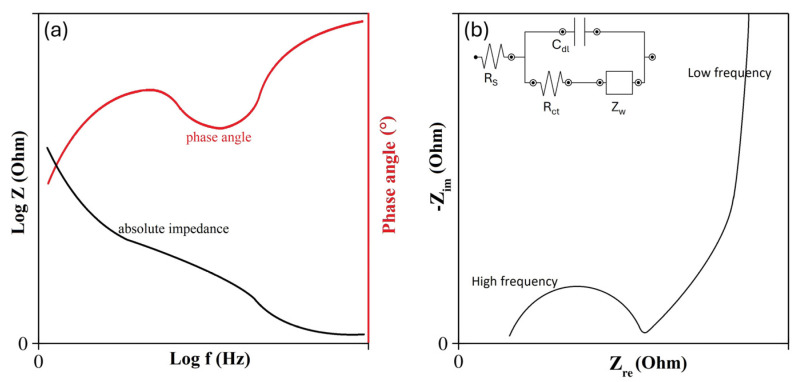
Schematic representations of (**a**) a Bode plot and (**b**) a Nyquist plot, measured with electrochemical impedance spectroscopy (EIS).

Electrochemical impedance spectroscopy (EIS) characterizes the capacitive performance of a material and determines the contribution of the electrodes and electrolytic processes. The impedance data are collected by applying an alternating potential at a small amplitude (e.g., ±5–±10 mV) over a wide range of frequencies (e.g., from 0.01 to 100 kHz). At higher frequencies, SCs become pure resistance, indicating that the ions of the electrolyte cannot penetrate the micropores at high frequencies. The pseudo-charge transfer resistance can be observed in frequencies ranging from high to low (10^4^ to 1 kHz), while, at very low frequencies (<1 kHz), pure capacitive behavior is the key feature of the impedance diagram. The results can be depicted in a Bode plot (Figure 10a) where the phase angle is expressed as function of the frequency, or in a Nyquist plot (Figure 10b) to show the imaginary and the real parts of the cell impedances. EIS can also be used to evaluate different mechanisms, e.g., charge-transfer, mass transport, and charge-storage [63,65,73,74,75].

## 5. Components of Supercapacitors

### 5.1. Electrode Materials

The charge-storage capabilities of SCs are highly dependent on the applied electrode material; thus, newly designed electrode materials with improved performance are a focus of SC development. Ideally, the electrodes should have a high electrical conductivity, adequate electrochemical and temperature stability, large electrochemically active surface area, and good surface wettability by the electrolyte. Recyclability and cost-effectiveness are also important aspects to be met [76,77]. There are different factors that contribute to the electrochemical performance of electrode materials, for instance the surface morphology, pore structure, and specific surface area. In this chapter, the most commonly used electrode materials are discussed, while current advances in the application of novel materials are also explored.

#### 5.1.1. Carbon-Based Materials

Carbon-based electrode materials are attractive for energy storage devices, as they provide high chemical/thermal stability and excellent conductivity, and are cost-effective [78]. Nanostructured carbon materials are used in EDLCs. In practice, not all of the surface area is fully accessible to the interaction of the electrolyte and the electrode; thus, the electrochemically accessible area can be called the electrochemically active surface area [79]. A large electrochemically active surface area, as well as the pore-size distribution, pore-shape, electrical conductivity, and functional groups of the surface are the main factors that determine their electrochemical performance [9,80,81]. These materials show a nearly rectangular-shaped cyclic voltammogram (Figure 8a).

Activated carbon (AC) is a widely used SC electrode material due to its high SSA and relatively low cost. Appropriate synthesis methods provide sp^2^-hybridized porous 3D carbon structures. Their pore structure can be characterized by micropores (<2 nm), mesopores (2–50 nm), and macropores (>50 nm). These are usually obtained from carbon-rich precursors through heat-treatment under an inert atmosphere (carbonization) and activation resulting in the formation of pores. Their precursors can be obtained from natural renewable natural resources, e.g., coconut shell, fossil fuels, pitch, coal, bamboo, or synthetic organic molecules, e.g., polymers, thiourea and formaldehyde [76,82]. Carbonization is used to produce amorphous carbon (by thermal chemical conversion), while activation results in a large surface area. The activation can be physical (in oxidizing atmosphere: CO_2_, H_2_O), or chemical (alkalis or acids) [79]. The application of different hard (zeolites, silicates) or soft (surfactant micelles) templates with an inherent porous structure can help to construct ACs with a 3D framework [76].

**Figure 11:**
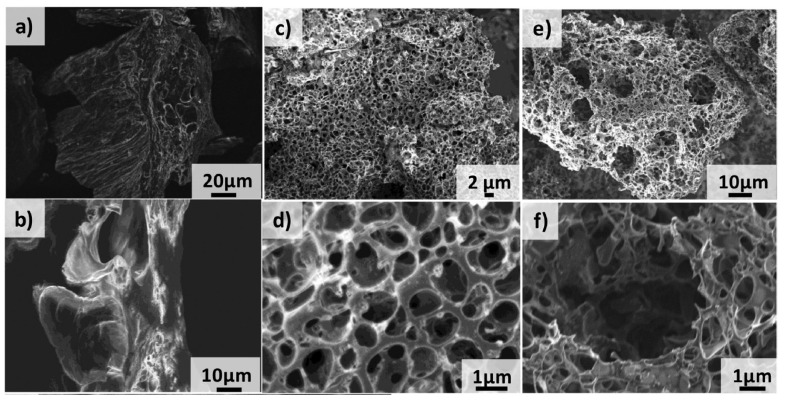
SEM micrographs of the hierarchical activated carbon material derived from the pyrolysis of fig fruit at different temperatures. (**a**,**b**) Inner part of the fruit after stabilization at 250 °C in air. ((**c**,**d**) and (**e**,**f**)) Inner part after pyrolysis with KOH chemical activation at 700 and 900 °C, respectively, showing the formation of interconnected meso- and macro-pores within the material [84]. Copyright Wiley-VCH GmbH. Reproduced with permission.

Kanokon et al. [83] reported on carbon nano sheets obtained from stinging nettle, exhibiting a surface area of 800 m^2^/g with a pore size below 3 nm and reaching a C_S_ of 27.3 F/g at 5 mV/s, an energy density of 0.06–0.95 Wh/kg, and a power density of 20.9–26.7 W/kg at 0.05 A/g. The anodic peak current of CV measurements was found to be linearly dependent on the square root of the scan rate, indicating that the electrochemical process is limited by the rate of diffusion. Further increasing the surface area (2000 m^2^/g), Ba et al. [84] obtained porous carbon from fig fruit (Figure 11), displacing the C_S_ of 340 and 217 F/g at 0.5 and 20 A/g, with an exceptionally high stability and 99% capacitive retention after 10,000 cycles. Wang et al. [85] applied a novel approach, using dual-aerogels of resorcinol-formaldehyde and polyvinyl alcohol as precursors to prepare hierarchical porous carbon. The produced carbon reached a specific surface area of 2076 m^2^/g, with a specific capacitance of 320 F/g at 1 A/g, while the assembled symmetric capacitor had an energy output of 59.8 Wh/kg at 350 W/kg in organic electrolytes with a potential range of 3.5 V. The long-term cycle stability of this product was found to be superior, showing 93.8% after 10,000 cycles. Ma et al. [86] synthesized N,O-doped carbon nanoplatelets with a high SSA, produced from covalent organic frameworks via high-temperature pyrolysis. The precise design of the structure and active centers resulted in a specific capacitance of 630 F/g and a high energy density of 15.28 Wh/kg at 352.5 W/kg in a 6M KOH electrolyte. Another carbon-based composite, a hierarchical structure of NiCoS_4_/N-doped carbon nanofiber electrode, was prepared by Abdel-Salam et al. [87]. The porosity of the structure allowed a specific capacitance of 754.4 F/g (at 1 A/g), while the constructed asymmetric SC showed an energy density of 52.5 Wh/kg at a power density of 1313.8 W/kg, with a moderate capacitance retention of 72% after 3000 charge/discharge cycles. Zhu et al. [88] prepared a 3D carbon-based MoO composite electrode via in situ growth and phase transformation, obtaining a specific capacitance of 411 F/g in a NaSO_4_ electrolyte, and retaining an excellent capacity of 94.1% after 5000 cycles. The carbon prevented the MoO_2_ from stacking, thereby minimizing the volume expansion of the electrode during the electrochemical processes, which is one of the most common drawbacks of pseudocapacitor electrodes.

It is worth mentioning that an excessive activation process will lead to higher pore volumes, which ultimately reduces the material density and conductivity and thereby lowers the power capability, while a larger specific area increases the risk of electrolyte decomposition. Therefore the optimization of these parameters is highly recommended [65]. Most of the commercially available devices are constructed with activated carbon electrodes and organic electrolytes, utilizing wider operating potential ranges [89].

**Figure 12:**
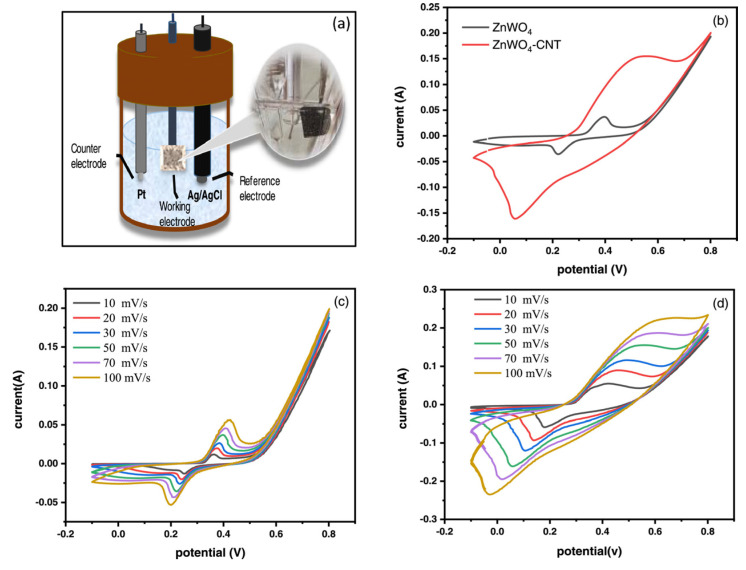
(**a**) The setup of SC configuration, (**b**) CV curves of ZnWO_4_/Nickel foam (NF) and ZnWO_4_-CNT/NF at 50 mV/s, (**c**) ZnWO_4_/NF, and (**d**) ZnWO_4_-CNT/NF at various scan rates. Reprinted from [102] with permission from Elsevier.

Carbon nanotubes possess fascinating electrochemical properties, such as high specific capacitance, stability under high current loads, and low internal resistance; therefore, CNTs make excellent polarizable electrodes. They can be deposited efficiently by a catalyst-supported chemical vapor deposition technique (CCVD) [90]. Many studies have discussed both single-wall nanotubes (SWCNTs) [91,92] and multi-wall nanotubes (MWCNTs) [93,94] as electrochemical SC electrodes. The surface of CNT electrodes is usually mesoporous. The specific capacitance of CNTs is greatly influenced by their purity and their morphology [95]. Many efforts are focused on developing a dense, aligned CNT forest that could increase their capacitance retention at higher currents by tuning the distance between the tubes [96,97,98]. An advantage is that CNTs can be grown on a conductive substrate without the need for a binder, thereby minimizing the contact resistance. The specific capacitance of purified CNTs is in a range from 20 to 80 F/g due to their hydrophobic property; however, it can be further improved by a subsequent oxidative process to about 130 F/g [99]. The electrochemical performance of CNTs can be improved by combining them with pseudocapacitive materials, thus creating composite electrodes. NiO–MnO_2_-coated MWCNT electrodes were fabricated by Hwang et al. [100] by a simple chemical precipitation method. A specific capacitance of 193.50 F/g (with 5 mV/s scan rate) could be achieved in a 6M KOH electrolyte. The shapes of the measured GCD curves were symmetrical triangles, indicating a perfect EDLC behavior and reversible charge/discharge processes. Embedding ZnO nanorods on functionalized CNT by a chemical refluxing method is another approach to create nanocomposite electrode materials, achieving a specific capacitance of 189 F/g (1 mV/s), high power density of 2250 W/kg, and energy density of 10.7 Wh/kg [101]. Maghadam et al. [102] synthesized a ZnWO_4_-CNT composite electrode by a simple hydrothermal method. The electrode exhibited a 4552 F/g specific capacitance (at 1 A/g) in a 3M KOH electrolyte, retaining an excellent stability of 92% after 3000 cycles. Using this electrode, a symmetric capacitor was assembled with a capacitance of 320 F/g (at 1 A/g), in the potential window of 0…1.3 V, and with a cycling stability of 78% (after 3000 cycles). Figure 12 shows the setup of electrochemical measurements and CV curves of the synthesized materials with different configurations and at different scan rates. Mandal et al. [103] prepared a carbon-based composite electrode using activated carbon and functionalized multi-walled carbon nanotubes. In a 3M KOH electrolyte, the electrode exhibited a maximum capacity of 395 F^/g^ (at 5 mV/s), and 372 F/g (at 60 A/g) with a capacity retention of 89% after 5000 cycles. An energy density of 25.31 Wh/kg and a corresponding power density of 75.27 kW/kg was achieved in their case.

Graphene is a structural arrangement of sp^2^-bonded carbon atoms in a honeycombed single layer. The arrangement of the C atoms enables tuning its properties within broad ranges. Graphene is a potential SC electrode material due to its high cyclic life, excellent chemical and thermal properties, short diffusion distance due to its thinness, and high availability of functional groups. Graphene has a specific surface area of around 2630 m^2^/g, which much higher than that of CNTs [104,105].

**Figure 13:**
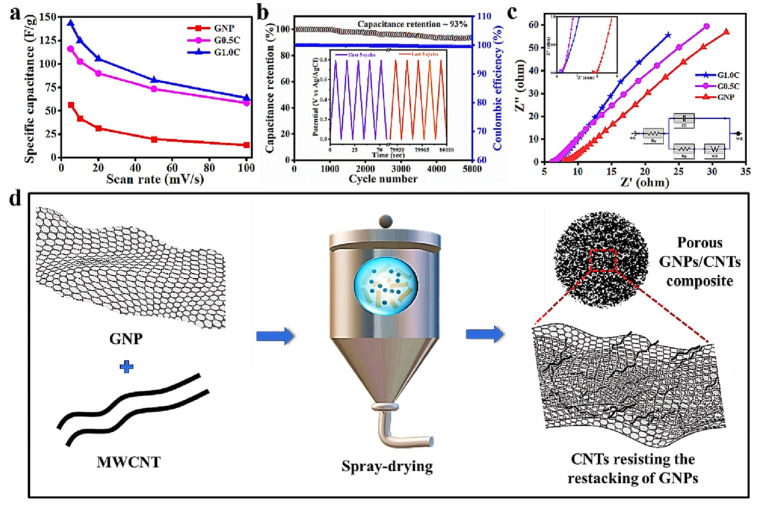
(**a**) Specific capacitance at different scan rates for graphene nanopowder (GNP), Graphene 0.5 wt.% CNT (G0.5C), and Graphene 1 wt.% CNT (G1.0C); (**b**) cyclic stability of G1.0C electrode; (**c**) Nyquist plot with equivalent circuit diagram; (**d**) schematic illustration of GNP/CNT porous composite engineered with CNTs spacer. Reprinted from [113] with permission from Elsevier.

Graphene-based SCs were reported with a C_S_ of 75 F/g with ionic liquid electrolytes, C_S_ of 135 F/g in aqueous medium, and 99 F/g in organic electrolytes [106,107,108]. On the other hand, graphene suffers from irreversible capacity loss as a consequence of re-stacking of the graphene sheets, which also reduces the initial Coulombic efficiency. The re-stacking occurs due to the van der Waals interaction between the adjacent sheets, reducing the surface area and lowering its energy density [109]. The re-stacking can be avoided by developing graphene-containing composites, e.g., graphene–CNT or graphene–metal oxides. Metal oxides prevent graphene from agglomeration and re-stacking, and increase the available surface area. The experimentally obtained capacitance in graphene/metal oxide composites is higher than the sum of the calculated capacitances for each material individually. As an example, RuO_2_-supported graphene was prepared by a hydrothermal method, reaching a specific capacitance of 551 F/g at 1 A/g in a 1 M H_2_SO_4_ electrolyte [110]. Regular symmetric triangular-shaped GCD curves were obtained in this case, verifying the superior capacitive behavior. The graphene/RuO_2_ electrodes were found to retain a higher capacitance of 94.3% as compared to the single graphene electrode (87%) after 2000 cycles. Mandal et al. [111] reported a ternary ZnFe_2_O_4_/graphene/activated carbon hybrid electrode prepared by the hydrothermal method. The graphene increased the specific capacitance of activated carbon to 176 F/g (at 1 A/g), while, with the addition of ZnFe_2_O_4_, it reached 533 F/g (at 1 A/g). The fabricated symmetric SC exhibited 5.42 Wh/kg (at 1A/g) and 4992 W/kg (at 10 A/g), with an excellent capacitance retention of 96% after 10,000 cycles. This enhancement can also be obtained by combining graphene with other materials, such as graphene/CNTs, graphene/polyaniline, and Pt/exfoliated graphene [3]. The chemical doping of graphene with electron donors and acceptors is another way to improve the electrochemical properties of graphene-based electrodes. A capacitance of 320 F/g was reported for highly nitrogenated graphene oxide [112]. The electrochemical behavior of graphene can also be improved by the addition of CNTs. Pandit et al. [113] prepared a graphene nanopowder (GNP)/CNT composite electrode by a facile and cost-effective spray drying technique (Figure 13). MWCNTs were used to inhibit the agglomeration of GNPs, reaching a specific capacitance of 143 F/g (at 5 mV/s) and a 93% cyclic stability after 5000 cycles.

Carbon aerogel is an ultra-light synthetic form of carbon containing nano-sized covalently bonded particles. They possess a porosity of over 50% and a SSA between 400 and 1000 m^2^/g. A flexible cellulose nanofibril/reduced graphene oxide (GO) composite aerogel was reported [114] with an outstanding compressibility (with a strain of 80%) and electrochemical performance of 31.2 mF/cm^2^ at 0.3 mA/cm^2^ in a PVA/H_2_SO_4_ gel electrolyte. The GO acted as a skeleton to prevent the shrinkage of nanocellulose during its carbonization and ensure excellent mechanical strength. The achieved porosity and hydrophilicity of the electrode material provided rapid ion diffusion at the electrode/electrolyte surface. Another study [115] reported on 2,2,6,6-tetramethylpiperidine-1-oxyl radical (TEMPO)-oxidized cellulose nanofibril (TOCN)- and graphene oxide (GO)-based carbon aerogels, presenting a specific capacitance of 398.47 F/g at a current density of 0.5 A/g. These compressible electrodes are promising electrode materials for flexible wearable electronic applications.

#### 5.1.2. Transition Metal-Based Compounds

Different types of transition metal-based compounds can be utilized as active materials of SCs, such as oxides, hydroxides, sulfides, phosphides, and nitrides. Their energy storage mechanism is usually based on reversible redox reactions; thus, they are ideal pseudocapacitive materials.

**Figure 14:**
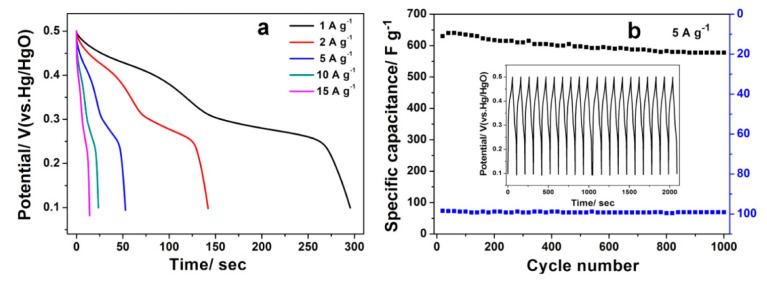
(**a**) Discharge curves of the Co_3_O_4_-modified electrode at different current densities (1…15 A/g), (**b**) specific capacitance and coulombic efficiency (η) of the Co_3_O_4_-modified electrode as a function of cycle number. Reprinted (adapted) with permission from [117]. Copyright (2014) American Chemical Society.

Transition metal oxides are preferred due to their multiple redox states. These materials typically show the cyclic voltammogram shapes shown in Figure 8b,c. The R_ES_ of RuO_2_ is very low, thus possessing a higher E_d_ and P_d_ than EDLCs; however, its high cost and toxicity limit its use in SC devices [116]. Beside RuO_2_, cobalt oxide, nickel oxide, vanadium oxide, manganese oxide, and copper oxide have also been thoroughly investigated as pseudocapacitive electrode materials. Nanostructured hollow Co_3_O_4_ prepared by a simple aqueous method was reported to achieve a specific capacitance of 820 F/g (with 5 mV/s) in a 6M KOH electrolyte [117]. The two discharge flats of each GCD curve (Figure 14a) corresponded to the two pairs of redox peaks measured in the CV curves, confirming the pseudocapacitive behavior of the electrode. Less than a 10% loss in the SC value was observed after 1000 cycles in their case (Figure 14b). Rui et al. prepared hydrated V_2_O_5_ single crystalline nanosheets by a sol-gel technique. In an organic LiClO_4_-propylene carbonate electrolyte, the prepared electrodes showed a specific capacitance of 219 F/g (with 1 A/g), and an energy density of 122 Wh/kg at a power density of 1.1 kW/kg. A capacitance retention of 81% was found after 1000 cycles [118]. Thalji et al. [119] prepared an intercalation pseudocapacitive electrode by growing Co-doped tungsten suboxide (W_18_O_49_) nano-needles onto carbon cloth. Due to the distorted structure of W_18_O_49_, and increased O vacancies, accelerated ion diffusion was made possible, resulting in a specific capacitance of 792 F/g at 1 A/g in a 1.0 M AlCl_3_ aqueous electrolyte, exceeding the capacity of the undoped W_18_O_49_. The anodic and cathodic peaks of the CV curves confirmed the typical intercalation-type pseudocapacitive behavior. Additional valence states can be introduced by using ternary (MnMoO_4_, NiCo_2_O_4_) [120] and quaternary (NiCoMoCuO_x_, NiCoMoZnO_x_) [121] metal oxides, thereby improving the achievable electrochemical characteristics. A NiCo_2_O_4_@Ni-Co layered double hydroxide structure was prepared by Chen et al. [122] on flexible carbon fiber cloth, which exhibited a 4.902 F/cm^2^ (at 2 mA/cm^2^) specific capacitance. The prepared electrode combined with a negative AC electrode reached a specific capacitance of 181.5 mF/cm^2^, and an energy density of 0.859 mWh/cm^3^ at the power density of 2 mA/cm^3^.

Sulfides of manganese (MnS), nickel (NiS, Ni_3_S_2_), copper (CuS), and other transition metals are also well-known pseudocapacitive materials. For this purpose, RuS_2_ nanoparticles were produced by Krishnamoorthy et al. [123] by an aqueous sonochemical method. The prepared electrode showed a maximum capacitance of 85.41 F/g at 0.5 mA/cm^2^. Symmetric charge and discharge curves were measured with GCD, indicating ideal capacitive properties. An assembled symmetric SC was assembled that presented 17 F/g (at 0.1 A/g), and excellent cyclic stability (96.15% after 5000 cycles). An intercalation pseudocapacitor electrode with an enhanced specific capacitance was prepared by Lichchhavi et al. [124] by coating Bi_2_S_3_ nanoflakes onto a Ni foam current collector without the need for a binder. The electrode exhibited superior electrochemical performance with a specific capacity of 1906 Ah/g (at 5 A/g), and with a cyclic stability of 97.22% after 2000 cycles, owing to the improved adhesion and structural stability of the Bi_2_S_3_/Ni foam. Similar to metal oxides, ternary metal sulfides possess a higher electronic conductivity and higher electrochemical activity than single-component metal sufides. For example, CuCo_2_S_4_ in many different forms have also been investigated. Using a 2M KOH electrolyte, 3D nanorods exhibit 515 F/g (at 1 A/g), and solvothermal-synthesized mesoporous particles have a 752 F/g (at 2 /g), while microwave-synthesized mesoporous nanoparticles show a 580 F/g (at 1 A/g) [125].

Transition metal nitrides and phosphides exhibit similar electrochemical properties. Ni_2_P nanoparticles, synthesized by a solvothermal method, showed a specific capacitance of 668.7 F/g (at 1 A/g), while asymmetric SCs with N_i2_P as positive and activated carbon as negative electrodes exhibited 371.1 F/g (at 1 A/g), reaching an energy density of 64.6 Wh/kg at a power density of 1029 W/kg [126]. A mesoporous gallium nitride (GaN) membrane was prepared by Wang et al. [127], and showed a specific capacitance of 23.11 mF/cm^2^ (at 0.5 mA/cm^2^). Similarly to carbon-based materials, the preparation of composite materials consisting of transition metal-based compounds can further enhance their electrochemical behavior [128].

By creating composite electrodes from different transition metal-based compounds, or combining them with other types of materials, their supercapacitive behavior can be further improved. Zhu et al. [129] constructed NiCo_2_S_4_@CeO_2_ microspheres with a structure consisting of nanosheets or nanoneedles. The introduction of CeO_2_ modified the compact structure of NiCo_2_S_4_, resulting in a porous structure with a higher SSA. A high specific capacitance of 1263.6 F/g was achieved with a retention of 81.1% after 10,000 cycles, owing to the facilitated electrolyte diffusion and the increased amount of redox active sites within the porous structure. The GCD curves showed nonlinear shapes due to the redox reaction, revealing typical pseudocapacitive characteristics. The poor electrical conductivity of FeO_x_ was enhanced by the fabrication of an FeOx/polypyrrole (Ppy) composite electrode via electrodeposition [130] on the surface of a graphite current collector. The composite film exhibited a surface capacitance of 2.0 F/cm^2^ (at 1 mA/cm^2^), with an excellent cycling stability of 105.6% after 10,000 GCD cycles. The FeO_x_–Ppy composite exhibited a longer discharge time and a higher discharge capacitance than pure PPy and FeO_x_ individually. In the low-frequency region of the EIS measurement, the slope of FeO_x_–Ppy was found to be higher than those of FeO_x_ and PPy, suggesting a higher ion diffusion rate of the composite, while, in the high frequency region, FeO_x_–Ppy exhibited a smaller equivalent series resistance (*R_ES_*) and charge-transfer resistance (*R_ct_*) compared with PPy and FeO_x_.

#### 5.1.3. Conducting Polymers

Besides transition metal-compounds, redox-active conducting polymers (CPs) have also attracted scientific attention as pseudocapacitive active materials. A large theoretical specific capacitance, good electrical conductivity, recyclability, relatively low cost, and the offer of large-scale production are their main advantages [131]. Their conduction is made possible through a conjugated bond system along the polymer backbone. They can be synthesized by chemical/electrochemical oxidation of the monomer [132]. Functionalization of the polymers allows for tuning the oxidation and reduction potentials to optimize the operating voltage of SCs. Due to their flexibility, they can be applied in wearable electronics as well [133,134]. Their major drawback is their poor cycle stability as a consequence of swelling/shrinkage of the electrodes during charging/discharging processes. In order to overcome this issue, binary or ternary CP-based composites are usually synthesized, combining them with transition metal-based compounds or carbonaceous materials [135]. The polarizability of the conducting polymers is determined by the doping properties. Conducting polymers can be doped with (counter) anions when oxidised (p-doping) and with (counter) cations when reduced (n-doping) [132].

**Figure 15:**
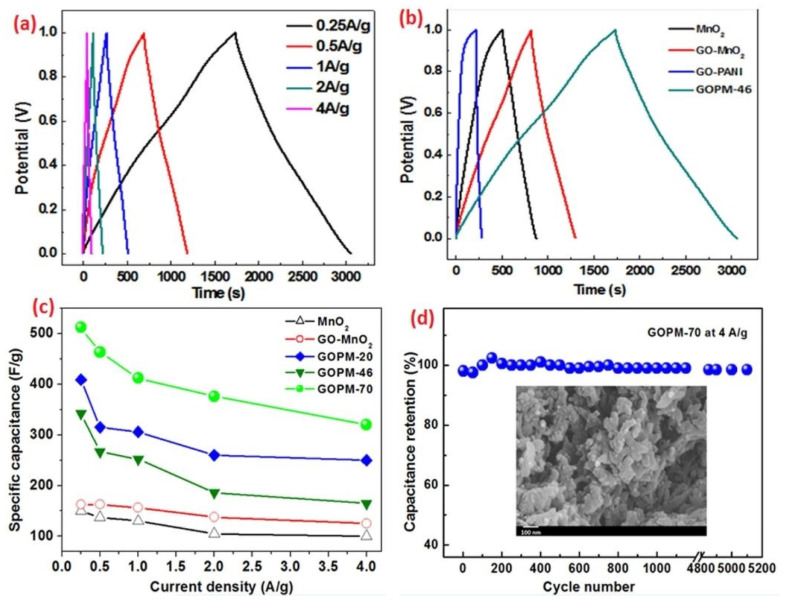
Galvanostatic charge/discharge curves of (**a**) graphene oxide (GO)-PANI-MnO_2_ (GOPM-46, with 46 wt.% MnO_2_) at different current densities; (**b**) MnO_2_, GO-MnO_2_, GO-PANI, and GOPM-46 at 0.25 A/g current density; (**c**) specific capacitance curves of MnO_2_, GO-MnO_2_, and GOPM composites at different current densities; (**d**) capacitance retention of GOPM-70 (with 70 wt.% MnO_2_) over cycling times [139]. Licensed by CC BY 3.0 (https://creativecommons.org/licenses/by-nc-nd/3.0/, accessed on 12 January 2024).

The most commonly studied supercapacitive polymers are polypyrrole, polyaniline, polyacethylene, and derivatives of polythiophene [136]. Polyaniline has a high electroactivity, controllable electrical conductivity, and high specific capacitance (400–500 F/g, at pH < 7) [137,138]. The deformation of the polyaniline as an electrode can be overcome by coating it onto metal oxides/carbon materials. Han et al. [139] prepared a ternary composite material by fastening MnO_2_ nanorods onto the surface of graphene oxide nanosheets via a polyaniline layer. The composite electrode exhibited a specific capacitance of 412 F/g (at 1 A/g, Figure 15c), and a very good electrochemical stability of 97% (Figure 15d) after 5100 cycles (at 4 A/g). Polypyrrole is known to be more flexible in regards to electrochemical applications; thus, it has been a subject of much research on the development of SC electrodes. However, it can only be p-doped, thus restricting its application to a cathode material. It has a greater density, thereby limiting the access to the interior structure for dopant ions, and reducing the gravimetric capacitance [132,140]. When incorporating reduced graphene oxide into polypirrole, the hybrid composite material can reach a specific capacitance of 424 F/g (at 1 A/g), combining both EDLC and faradaic mechanisms [141]. EIS measurement revealed that the introduction of reduced graphene oxide lowered the charge-transfer resistance of the electrode. Polythiophene can be n- and p-doped as well; however, the n-doped form suffers from many disadvantages compared to the p-doped form, e.g., lower gravimetric capacitance, poor conductivity, and low stability when exposed to oxygen and water, which results in self-discharge. To overcome these issues, polythiophene derivatives can be used. PEDOT is a popular derivative with a high conductivity and high operating potential range of 1.4 V, as well as a specific capacitance of 133 F/g [132]. With the addition of MnO nanoflakes via electrodeposition, and a shield layer of PEDOT fabricated on the surface of a flexible activated carbon cloth, a hierarchical nanostructured composite electrode was prepared by Akbar et al. [142]. An exceptional capacitance of 1882.5 mF/cm^2^ (at 1 mA/cm^2^) was achieved in 1.5 M aqueous LiCl electrolyte. The fabricated asymmetric SC demonstrated a wide voltage window of 1.8 V and an excellent cycling stability of 94.6% after 10,000 GCD cycles.

#### 5.1.4. Novel Materials

The efforts to increase the efficiency of SCs have led to the emergence of new types of electrode materials. Metal–organic frameworks (MOFs) and MXenes are the most notable representatives, and were first developed in the last 10–30 years.

**Figure 16:**
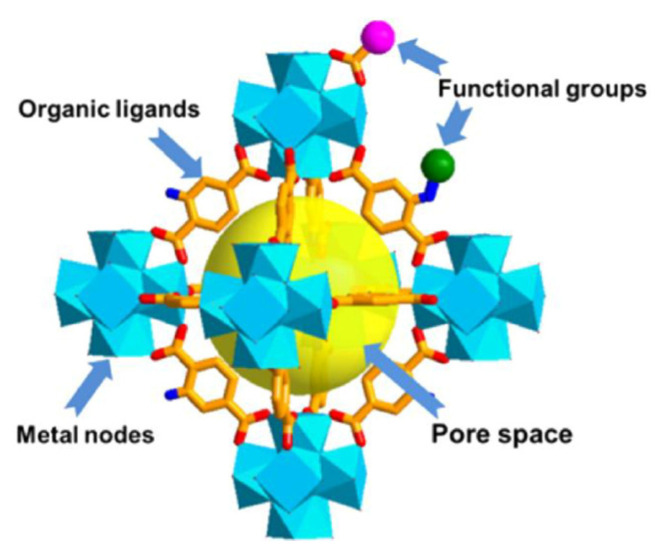
Schematic representation of functional MOF structures. Reprinted from [150] with permission from Elsevier.

MOFs are new crystalline materials, with a structure in which organic ligands are repeatedly coordinated to metal cations via strong chemical bonds, forming from one-dimensional to three-dimensional frameworks (Figure 16). The organic units are divalent or polyvalent carboxylates, while the metal containing units can be ions of transition metals (Zn, Co, Cu, Ni), alkaline earth elements (e.g., Sr, Ba), or p-block elements (e.g., In, Ga) [143]. As an example, MOF-5 has a structure of linked Zn_4_O tetrahedrons via 1,4-benzenedicarboxylate organic ligands with an interconnected pore structure (pore diameter of 1.2 nm) [144,145]. These structures efficiently connect the field of nanotechnology with different energy storage applications, owing to their inherent porosity, controllable morphology/topology, and electroactive properties. As well, MOFs are ideal templates and precursors for the preparation of porous carbon and transition metal-based compounds. The pore structure, surface area, and electrical conductivity of MOFs can be tuned by the selection of metal ions and different organic linker units. The capacitive performance of MOFs can be further improved by combining them with different carbonaceous materials (e.g., rGO, graphene, CNT) to improve their conductivity [146]. A Co-based MOF was synthesized by Lee at al. [147] to produce a SC electrode material. The electrode exhibited a specific capacitance of 206.76 F/g and an energy density of 7.18 Wh/kg (both at 0.6 A/g). Yang et al. [148] prepared a layered two-dimensional Ni-based MOF via an aqueous method that reached specific capacitances of 1127 F/g (at 0.5 A/g) and 668 F/g (at 10 A/g) and a maximum power density of 1750 W/kg while retaining over 90% of its performance (after 3000 cycles). Qian et al. synthesized an interconnected macro-microporous Cu-based carboxylate MOF via a monodentate-ligand-assisted method. By applying a thermal transformation, the derived microporous carbon served as a SC electrode in sulfuric acid, exhibiting specific capacitances of 214 F/g (at 5 mV/s) and 236 F/g (at 0.5 A/g) [149].

MXenes are a new class of two-dimensional materials that was first developed in 2011 [151]. They consist of a few atomic layers of transition metal carbides, nitrides, or carbonitrides, with a general formula of M_n+1_X_n_T_x_, where M represents a transition metal (Ti, Mo, Cr, Nb, V, Sc, Zr, Hf, or Ta), X stands for carbon or nitrogen, T_x_ is the surface termination such as hydroxyl, oxygen, or fluorine, and n is an integral number that is usually between 1 and 3. They are produced by selective etching of the element A from a 3D layered MAX phase, where A is an element of group 13 or 14. MXenes are attractive materials for the electrodes of energy storage applications due to their structural properties, i.e., an inner transition metal carbide layer enabling efficient electron transportation and a metal oxide-like surface layer acting as an active site for fast redox reactions [152]. To date, more than 20 MXenes have been synthesized and have shown superior specific capacitance due to the reversible intercalation of metal cations (e.g., Na^+^, K^+^, Mg^2+^, Al^3+^) [153]. A flexible paper electrode was developed by Fu et al. [154] based on layered Ti_3_C_2_T_x_. The electrode exhibited a specific capacitance of 892 F/cm^3^ (at 2 mV/s) and a long-term cyclic performance without capacity loss (after 10,000 cycles). Syamsai et al. [155] produced Ta_4_C_3_ MXene by etching Al from its MAX phase, and it showed a maximum capacitance of 481 F/g (at 5 mV/s) and a good cyclic retention of 89% (after 2000 cycles) in a H_2_SO_4_ electrolyte as a SC electrode. Hu et al. [156] uniformly coated a conductive carbon textile cloth with Ti_3_C_2_T*_x_* MXenes to create a flexible electrode that exhibited a surface capacitance of 362 mF/cm^2^ and an excellent cyclability. These studies support that MXenes are ideal materials for energy storage applications in wearable smart electronics. One of their disadvantages is the restacking of the MXene layers, although this could be prevented by combining them with MOFs to make composite electrode materials. Adil et al. [157] prepared a FeCu bimetallic MOF–MXene composite electrode in a binder-free synthesis route, thereby shortening the ion/electron diffusion pathways, improving the electroactive sites, and preventing the restacking of MXene layers. When combining MXenes with PANI via in situ polymerization, a composite fiber-shaped electrode was reported [158] to be prepared, with a high surface capacitance of 510 mF/cm^2^ and with a 94.4% capacitance retention when the current density was increased. The GCD curves demonstrated symmetrical triangle shapes, implying capacitive charge-storage behavior and a high structural stability and reversibility.

**Figure 17:**
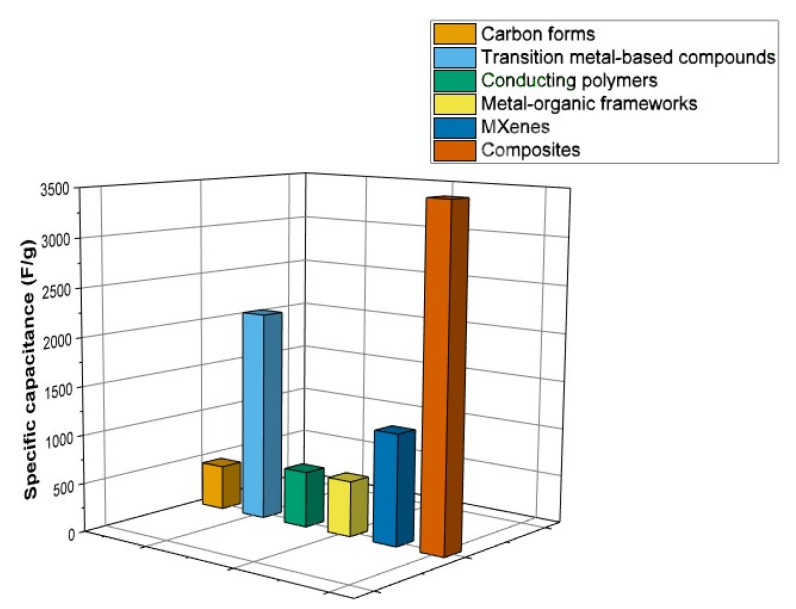
Comparison of the achievable specific capacitances of different supercapacitive materials [47,79,131,162,163,164,165].

It can be concluded that the different electrode materials exhibit different energy storage mechanisms. Figure 17 summarizes the achievable specific capacitances for these types of materials. As mentioned earlier, the electrochemical behavior of the electrodes can be improved by applying composite materials. The highest charge-storage ability can be obtained by combining these materials in a (hybrid) composite electrode. Composite electrodes can be characterized by their better rate capability, cyclic stability, and enhanced mechanical and electrical properties [159,160]. Hybrid composite electrodes usually combine non-faradaic and faradaic charge-storage mechanisms in one electrode. The main motivation factor behind their development was the enhancement of the energy density, limited by EDLC, and the lower power density of pseudocapacitive materials, thus overshadowing these disadvantages [77,161]. However, in addition to the capacitance, other important parameters such as the reliability and life cycle of the electrode material need to be considered as well, as summarized in Table 2.

### 5.2. Current Collectors

A current collector is a passive component of SCs that provides a physical support for the electroactive material. Its main function is to act as a transporting medium between the electrode material and the external circuit, while additionally dispersing the heat generated within the cell [61,167]. The electrical resistance of current collectors and the interfacial resistance of the current collector/active material highly contribute to the total resistance (R_ES_) of the SC device. The requirements of current collectors are thermal/electrochemical stability, high electrical conductivity, low contact resistance (between active materials/current collector), and appropriate mechanical strength [76,168]. The current collector should be selected according to the applied electrolyte and the active material. Metal-based current collectors, e.g., stainless steel, nickel, copper, and aluminum are generally used in the form of foils, foams, or meshes. As a cost-effective solution with higher electrochemical and thermal stability, carbon-based materials such as carbon fiber, carbon paper, glassy carbon, and graphite plate/foil can also be applied. The greater flexibility of the paper/fiber/foil forms are ideal for use in commercial wearable electronics [169,170].

In alkaline medium, Ni-based metals (pure Ni, Inconel alloy), stainless steel, and carbon-based materials (carbon fabrics, carbon cloth, graphite paper) can be efficiently applied [171]. In the case of Ni, a small pseudocapacitance value, due to the presence of NiO/Ni(OH)_x_, can also contribute to the total capacitance of SCs [172]. However, in acidic medium, Ni tends to dissolve into the electrolyte. In strong acid-based electrolytes, corrosion-resistant Au metal foils, indium-tin-oxide (ITO), and carbon-based materials are the suitable choice [171,173]. The commercially available SCs are usually based on organic electrolytes and aluminum current collectors [168].

The problem of corrosion and dissolution of metal current collectors into the electrolyte can be prevented by depositing a protective coating, e.g., a thin layer of carbon, onto its surface [174]. The surfaces of current collectors can also be modified by etching, thus increasing their adhesion with the active material and maintaining a more stable operation [175].

### 5.3. Electrolytes

One of the major challenges in the development of SCs is an insufficient energy density (E_d_), which is proportional to the capacity and the square of the cell voltage (see Equation (3)). This means that increasing these two parameters can improve their energy storage capability. It can be seen that the cell voltage contributes to a larger extent, as it is proportional to the square of the voltage. The academic research on this subject usually aims to increase the capacity through the development of novel electrode materials. The other way is to increase the working potential of the SC device, which strongly depends on the electrochemical stability of the used electrolytes. This leads to the development of new electrolytes, and to the careful design of the SC cell, by tuning the interactions between the active electrode material and the electrolyte to improve their synergistic effect [176]. As an example, the pore size and the shape of the pores of carbon-based materials should match the size of the electrolytic ions in order to efficiently utilize the maximum energy storage capability of the electrode [40]. Different types of electrolytes can be used, with their characteristic electrochemically stable voltage range, to avoid the decomposition of the electrolyte and the building of pressure inside the device. Aqueous electrolytes can be operated in a range of 1.0–1.3 V, as the thermodynamic potential window of water is relatively narrow (water decomposition reaction occurs at around 0 V and 1.23 V vs. NHE at 1.0 atm and room temperature). In the case of organic electrolytes, their stability range is between 2.5 and 2.7 V, while ionic liquids have a potential window between 3.5 and 4.0 V. The stability of the electrode material within the applied potential range is an important criterion as well. In addition, the applied electrolyte contributes to the internal resistance, rate performance, operating temperature range, self-discharge, and toxicity of the SC device as well [176]. The commonly employed electrolyte materials can be classified into two main categories: liquid electrolytes and solid-state/quasi solid-state electrolytes.

#### 5.3.1. Liquid Electrolytes

Liquid electrolytes can be further classified into aqueous electrolytes, organic electrolytes, and ionic liquids. Each of these electrolyte materials has its own advantages and disadvantages. Aqueous electrolytes (electrolyte salt + water as solvent) are known to have high ionic conductivity but suffer from a limited working potential window. Organic electrolytes and ionic liquids can withstand higher potential values but possess a considerably lower ionic conductivity. The commercially available SCs usually use organic electrolytes, mostly based on acetonitrile and propylene carbonate solvents [177]. On the other hand, asymmetric hybrid SCs use aqueous electrolytes, considering the manufacturing issues [61,178].

Aqueous electrolytes generally consist of acidic (e.g., HCl, H_2_SO_4_, HNO_3_), basic (KOH, NaOH), and neutral (Na_2_SO_4_, K_2_SO_4_) solutions. The maximum ionic conductivity can be obtained by optimizing the concentration of the solution. The mobility of the ions is generally affected by the solvent viscosity and the size of the hydrated cations and anions in the electrolyte [179]. In the case of EDLCs, the reported specific capacitance values obtained in H_2_SO_4_ electrolytes are usually higher, while the R_ES_ values are usually lower than in neutral electrolytes [176,180,181]. The alkaline electrolytes are usually used for carbon-based electrodes, pseudocapacitors (e.g., Ni- and Co-based electrodes), and hybrid SCs. Among them, KOH is the most commonly used (with a high conductivity of 0.6 S/cm for 6M solution at 25 °C) [176]. However, there are some limitations of using aqueous electrolytes, e.g., operating at higher temperatures can lead to the evaporation of the electrolyte, while corrosion of the electrode materials can occur, especially with highly concentrated acidic/alkaline electrolytes [182].

In the case of organic electrolytes, the conducting salts are dissolved in organic solvents. The higher operating voltage window enables the storage of a larger amount of energy. In addition, when using organic electrolytes, cheaper materials (e.g., Al current collector) can also be safely used. A commonly used conducting salt is tetraethylammonium tetrafluoroborate (TEABF_4_), which is dissolved in acetonitrile or propylene carbonate [183,184]. The lower ionic conductivity is a consequence of the lower solubility and the lower dissociation degree of the salts in the organic solvents. Some of the disadvantages should also be taken into account when considering their application, e.g., flammability, toxicity, and a higher cost. Similarly to aqueous electrolytes, the ion size, viscosity, and ion–solvent interactions determine the electrolyte performance. The organic electrolytes are preferably used for EDLCs, reaching a usually lower specific capacitance value than in aqueous electrolytes, usually as a consequence of larger ionic sizes and lower dielectric constants. In this case, a smaller pore size of the carbon electrode materials may enhance the specific surface area, but can limit the accessibility of these pores for larger ions [176,185]. In the case of pseudocapacitors, Li-containing organic electrolytes are usually applied to facilitate the intercalation and deintercalation processes [186,187].

Ionic liquids (ILs) are organic molten salts, usually consisting of a large organic cation and an inorganic/organic anion with a melting point below 100 °C [188]. They have the advantage over organic electrolytes, as they are non-flammable and non-volatile and have a high chemical/thermal stability with the broadest operating voltage range (0–5 V). Thus, they can be applied as alternative electrolytes for SCs. Their major benefit is the high tuneability of the relevant physical and chemical properties (e.g., operating voltage window, R_ES_) by the high number of combinations of cations and anions. Their limitation, however, is their relatively low ionic conductivity compared to the other two types of electrolytes [189,190]. The reported ionic liquids used for SCs generally contain imidazolium, ammonium, sulfonium, or phosphonium cations and tetrafluoroborate (${BF}_{4}^{-}$), hexafluorophosphate (${PF}_{6}^{-}$), or dicyanamide (${DCA}^{-}$) anions [176]. The obtainable specific capacitance values are usually lower than those of the other two electrolytes, most likely due to the high viscosity of ILs. In order to reduce the viscosity, mixture solutions containing additional organic solvents have been reported as well [191,192,193].

#### 5.3.2. Solid-State/Quasi-Solid-State Electrolytes

The rapid development of wearable, portable electronic devices introduced the need for solid-state electrolytes, especially when flexibility is a necessary feature. These electrolytes serve both as an ionic conducting medium and as a separator. Their application offers several safety benefits, e.g., reducing the risk of leakage, corrosion, and flammability, while many of them can withstand mechanical stresses as well [194,195,196]. The majority of the solid-sate electrolytes developed for supercapacitive applications are polymer electrolytes. Two main groups can be distinguished: solid polymer electrolytes and quasi-solid-state gel-polymer electrolytes.

Solid polymer electrolytes (also known as dry polymer electrolytes) are the most commonly used solid-phase types, and they usually consist of a polymer matrix (e.g., polyethylene oxide, polyvinylidene, polyvinylpyrrolidone) and a conducting salt (e.g., LiClO_4_, LiCF_3_SO_3_) [197,198], without the use of any solvent. Thus, the transportation of the ions takes place in the polymer phase. Xu et al. [198] prepared a solid-state electrolyte containing poly(vinylidene fluoride) (PVDF)/lithium triflate (LiTf)/epoxy via dip-coating and vacuum curing, and used glass fiber as a separator. The SC exhibited a specific capacitance of 0.128 mF/cm^2^, with excellent mechanical properties and cyclic stability.

**Figure 18:**
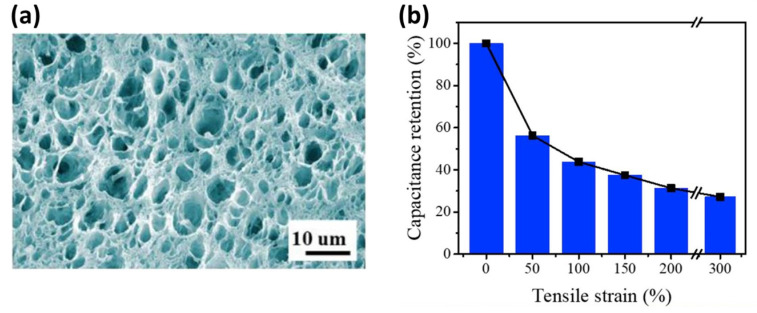
(**a**) Porous structure of a polyvinyl alcohol–tannic acid gel polymer electrolyte, (**b**) capacitance retention obtained from GCD curves with different tensile strains. Reprinted from [202] with permission from Elsevier.

The main drawback of solid-state electrolytes is, however, their low conductivity, which can be overcome by applying quasi-solid-state gel-polymer electrolytes (GPEs) [199,200]. GPEs consist of a polymer network which surrounds the liquid electrolyte and prevents the liquid from escaping. The used salts provide the conducting ions, while the solvent acts as the conducting medium; thus, GPEs provide a higher ionic mobility than solid polymer electrolytes [201]. For this reason, they have been extensively studied in recent years. Their major drawbacks are their relatively low mechanical strength and a narrow operating temperature range. Qin et al. [202] prepared a polyvinyl alcohol–tannic acid-based GPE (Figure 18a) that contained H_3_PO_4_ as a conducting electrolyte. The SC exhibited a specific capacitance of 102.7 F/g (at 50 mA/g), a good flexibility, and an adequate capacitance retention with high stretchability (Figure 18b), and that also possessed self-healing effects that could withstand a low temperature of −20 °C.

### 5.4. Separators

Separators, similarly to current collectors, are passive components of SCs without any contribution to the capacitive performance. However, their use as a barrier is essential to the operation of SCs, preventing any physical contact between the two electrodes, and thereby preventing any short circuit. Meanwhile, they allow the free flow of ions. Appropriate importance should be given to them, as low quality separators can deteriorate the performance of SCs by imposing additional resistance [61,203]. The separators should meet the following criteria: electrical insulation (with high dielectric constant), chemical/electrochemical inertness, ion transfer capability, appropriate porosity, mechanical resistance to pressure and volume changing occurring in the cell, and good wetting between the separator/electrolyte. Different materials can be used as separators, including glass fiber, ceramics, paper, and polymer membrane. Polymer-based separators, e.g., polypropylene, polypropylene-carbonate, and polyamide, as cost-effective materials with a porous nature and flexibility, are frequently applied [61,204]. Among them, polypropylene separators are commonly used in commercial applications. Macroporous poly(vinylidene fluoride) (PVDF) separators can be effectively applied in organic electrolytes, offering a high ionic conductivity and more electrolyte retention; however, their mechanical strength is lower compared to membranes with a higher density [205,206]. Today, the most commonly used dense separators are Nafion and sulfonated polyether ether ketone (SPEEK) membranes [204].

## 6. Conclusions

High performance SCs represent a cutting-edge technology in the field of energy storage. Unlike traditional batteries, SCs store energy either through the electrostatic separation of charges, or by fast redox reactions constrained to the electrode/electrolyte interface, allowing for rapid charge/discharge cycles. Researchers are usually focused on enhancing the energy density and overall performance of SCs to make them more competitive with conventional batteries. In this review, the different charge-storage mechanisms of EDLCs and pseudocapacitors are addressed, in parallel with the main electrochemical measurement methods. The advancements in materials science, particularly the exploration of novel electrode materials and electrolytes, that have played a crucial role in improving SC performance are the main focus of this paper. Crucial parameters such as the specific capacitance, energy, and power densities, as well as cyclability, are discussed along with examples of newly designed electrode materials and electrolytes. In EDLCs, besides the widely used activated carbon, nanostructured carbon materials such as CNTs, graphene, and carbon aerogels are extensively studied for their large surface area, excellent conductivity, and quick charge/discharge rates. Among pseudocapacitive materials, transition metal-based compounds (oxides, hydroxides, sulfides, phosphides) and conducting polymers (e.g., polypirrole, PEDOT) are commonly applied, while novel materials, such as TMOs and MXenes, hold future promises. The research findings discussed herein clearly indicate that the most effective supercapacitive electrode materials are the hybrid composites made from combinations of these materials, thereby merging their advantages. When considering the applied electrolytes, their role is usually underrated in the development of electrode materials; however, they are equally essential in determining the capacitive behavior of the cell. All of the main liquid electrolytes (aqueous, organic, and ionic liquids) have their own pros and cons, and it is essential to tune the special behavior of the applied electrode material and the electrolyte to achieve the desired functionality of the SC device. Furthermore, the passive components should not be neglected: the current collectors and separators. The material of the current collector should be selected based on the applied electrode and electrolyte to ensure safe and reliable application, while the quality of the separator is also essential to minimize the total resistance of the device. It can be concluded that the journey of SC development is dynamic, with each discovery and innovation bringing us closer to a sustainable and energy-efficient future.

## 7. Future Perspectives

The continued exploration of novel electrode materials such as 2D materials (MXenes) and MOFs hold potential in further enhancing the current performance of SCs. In addition, ensuring proper adhesion between the current collector and the electrode material is an important factor that requires further development for certain materials (e.g., activated carbon). Nanostructured electrode materials and nanostructural modifications of the surfaces of current collectors offer promising solutions in achieving more efficient electrochemical properties.

The pursuit of hybrid systems, combining the best features of EDLCs and PCs, has led to the development of hybrid composite electrodes and hybrid SCs. These systems aim to combine the benefits of different energy storage mechanisms, offering high energy and power densities along with an extended life cycle. Moreover, the hybridization of different energy storage technologies (e.g., SCs and batteries) can create systems which offer more promising application opportunities for consumer electronics such as electric vehicles and renewable energy systems. Besides, the development of flexible and stretchable SCs enables the opening of routes in wearable electronics as well. To ensure flexibility, the application of solid/quasi solid-state electrolytes is essential. The current results of studies on these electrolytes are promising, but future improvements are needed to find the optimal technological window to reach the desired mechanical strength, ionic conductivity, and thermal stability.

## Figures and Tables

**Table 1:** Merits and demerits of different characterization techniques [65].

Technique	Merits and Demerits
**CV**	**Merits**
	Degradation process evaluation is available Specific capacitance can be easily calculated Differentiate between EDLC and pseudocapacitor (PC)
	**Demerits**
	Shows only kinetic aspects, thermodynamics are neglected
**GCD**	**Merits**
	Capacitance can be easily calculated Differentiate between EDLC and PC
	**Demerits**
	Same triangular shape is shown for all double layer capacitive material
**EIS**	**Merits**
	Separately evaluate resistances in a system Specific capacitance can be calculated Differentiate between resistivity and inductive nature Non-destructive technique Relaxation time for recharging Informs on degradation behavior
	**Demerits**
	Evaluation is limited to small voltage Discrete behavior above 10^6^ Hz

**Table 2:** Comparison of features of different electrode materials for SC application [28,166].

Material Group	Material Types	Features	Importance for SCs
Carbon-based materials	Activated Carbon	High SSA, porous structure, physisorption	EDL capacitance, large amount of charge to be stored at electrode/electrolyte interface, high C_S_
	Carbon Nanotubes (CNTs)	Unique tubular nanostructure, high electrical conductivity, high SSA	EDL capacitance, high C_S_, high mechanical stability,
	Graphene	Excellent electrical conductivity, large SSA	EDL capacitance, high C_S_, fast charge/discharge rates
	Carbon aerogel	Interconnected highly porous network	EDL capacitance, efficient charge transport, high C_S_
Transitin metal based compounds	Transition metal oxides/hydroxides	Pseudocapacitive electrode materials, redox reactions	Fast, reversible faradaic charge-storage, C_S_ higher than with EDLCs
	Transition metal sulfides, phosphides, nitrides	Pseudocapacitive electrode materials, redox reactions, easy availability	Fast, reversible faradaic charge-storage, C_S_ higher than with EDLCs
Conducting polymers		Electrical conductivity, pseudocapacitive behavior	Redox charge-storage reactions, enhanced energy density
Metal organic frameworks		Porous materials, tunable structures, High SSA	Ability to incorporate redox-active metal centers
MXenes (Metal Nitrides and Carbides)		New class of 2D materials, high electrical conductivity, high SSA	Combination of EDLC and pseudocapacitance
Hybrid composite materials		Combine the advantages of different materials	Enhanced capacitance, rate capability and cyclic stability

## Data Availability

Some figures presented in this review were obtained from previously published papers. The proper copyright permissions are indicated in the caption of these Figures.
